# A Low-Fat/Sucrose Diet Rich in Complex Carbohydrates Reverses High-Fat/Sucrose Diet-Induced Corneal Dysregulation

**DOI:** 10.3390/ijms24020931

**Published:** 2023-01-04

**Authors:** Prince K. Akowuah, Carolina Lema, Rolando E. Rumbaut, Alan R. Burns

**Affiliations:** 1 College of Optometry, University of Houston, Houston, TX 77204, USA; 2 Children’s Nutrition Research Center, Baylor College of Medicine, Houston, TX 77030, USA; 3 Center for Translational Research on Inflammatory Diseases (CTRID), Michael E. DeBakey Veterans Affairs Medical, Houston, TX 77030, USA

**Keywords:** cornea, macronutrient, high-fat/sucrose diet, obesity, neutrophils, platelets

## Abstract

High-fat/sucrose diet feeding in mice causes loss of corneal nerve function and impairs corneal wound healing. While changing to a diet with a low fat/sugar composition and enrichments in complex carbohydrates mitigates the reduction in nerve function, it remains to be determined if it has an effect on corneal wound healing. In this study, 6-week-old C57BL/6 male mice were fed either a normal diet or a high-fat/sucrose diet for 20 weeks. A third group (diet reversal) was placed on a high-fat/sucrose diet for 10 weeks followed by a normal diet for an additional 10 weeks. A central corneal epithelial abrasion wound was created, and wound closure was monitored. Neutrophil and platelet recruitment was assessed by immunofluorescence microscopy. Mice fed the high-fat/sucrose diet-only had greater adiposity (*p* < 0.005) than normal diet-only fed mice; diet reversal markedly reduced adiposity. Following corneal abrasion, wound closure was delayed by ~6 h (*p* ≤ 0.01) and, at 30 h post-wounding, fewer neutrophils reached the wound center and fewer extravascular platelets were present at the limbus (*p* < 0.05). Diet restored normal wound closure and neutrophil and platelet influx in the injured cornea. These data suggest compositional changes to the diet may be an effective diet-based therapeutic strategy for maintaining or restoring corneal health.

## 1. Introduction

Dietary patterns involving high fat, sugar, and salt consumption, commonly known as Western diets, have fueled an increasing prevalence of obesity [1,2,3]. The global and U.S. prevalence of obesity has tripled over the last five decades [4,5]. The increasing prevalence of obesity increases the risk of high mortality rate systemic conditions, including metabolic syndrome, cardiovascular and kidney diseases, and Type 2 diabetes [6,7,8,9]. Obesity is the leading risk factor for Type 2 diabetes [10,11,12].

Diabetes is associated with corneal degeneration (keratopathy), a potentially sight-threatening condition involving impaired corneal wound healing, loss of corneal innervation and stromal transparency, and epithelial ulcerations [13,14]. Diabetic keratopathies have been well documented in both human and animal models of diabetes and prediabetes—up to 70% of people with Type 2 diabetes exhibit some form of keratopathy [14,15,16,17]. Although traditionally believed to be due to sustained hyperglycemia in diabetes, recent evidence in animal models of obesity suggests keratopathies can occur before hyperglycemia becomes established [18,19,20]. Using a diet-induced obesity mouse model, we have demonstrated impaired corneal wound healing and corneal neuropathy in obesity [18,19].

Furthermore, recent human studies confirm corneal nerve loss in individuals with severe obesity, including subsets of these individuals who lack sustained hyperglycemia [21,22]. Diabetic keratopathies are challenging to treat [23,24] and persist even when hyperglycemia is controlled [24,25]. Thus, it is crucial to explore therapeutic strategies for improving keratopathies in obesity before progression toward diabetes, a time during which disease arrest or reversal may still be possible.

Diet-based strategies have been the go-to in attempts to treat obesity due to their low-cost and ease of access compared to surgical and pharmacological techniques [26]. A typical strategy for treating obesity involves changes to the type and quantity of macronutrients in the diet. One such approach, diet reversal, involves switching from diets high in fat, sugars, and salts to diets traditionally high in complex carbohydrates and low in refined sugars and fat. This strategy mitigates various obesity-induced effects, including a reduction in body weight, improvement in insulin sensitivity, insulin resistance, glucose tolerance, systemic inflammation, restoration of circadian rhythm, and amelioration of memory deficits [27,28,29,30,31]. With respect to the cornea, rodent models of obesity show that diet reversal improves corneal nerve health [18,29]. However, obesity is also associated with impaired corneal wound healing [18] and it is unknown if diet reversal can correct this impairment. In this study, we used a diet-induced obesity mouse model to evaluate high-fat/sucrose feeding effects on corneal nerve function and corneal wound healing and the reversibility of these effects with a diet reversal strategy. We hypothesized that diet reversal would ameliorate reduced corneal nerve function and impaired corneal wound healing in mice fed the high-fat/sucrose diet.

## 2. Results

### 2.1. High-Fat/Sucrose Diet Feeding for 10 Weeks Causes Significant Adiposity and Impaired Insulin Metabolism

**Figure 1:**
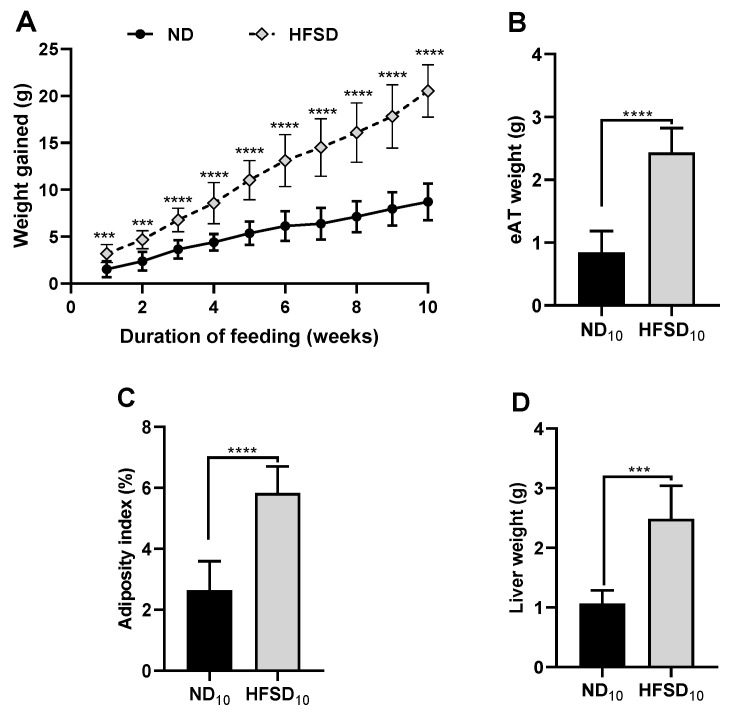
High-fat/sucrose diet feeding for 10 weeks causes significant adiposity and liver weight gain. (**A**) Cumulative weight change (**B**) Epididymal adipose tissue weight, (**C**) body adiposity index, and (**D**) liver weight in mice fed a normal diet or high-fat/sucrose diet (*n* ≥ 6 per group). Data expressed as means ± SD. ND_10_—10-week normal diet; HFSD_10_—10-week high-fat/sucrose diet; *** *p* ≤ 0.001, **** *p* ≤ 0.0001.

Mice fed the high-fat/sucrose diet gained more weight than those fed the normal diet (*p* < 0.003). An age-related increase in body weight was observed in normal diet-fed mice, which was exacerbated by high-fat/sucrose diet feeding in the high-fat/sucrose diet-only mice (Figure 1A). Mice fed the high-fat/sucrose diet had increased eAT weight (*p* < 0.0001), greater visceral adiposity index (*p* < 0.0001) (Figure 1B,C) and increased liver weight (*p* < 0.001; Figure 1D) compared to mice fed the normal diet only.

**Figure 2:**
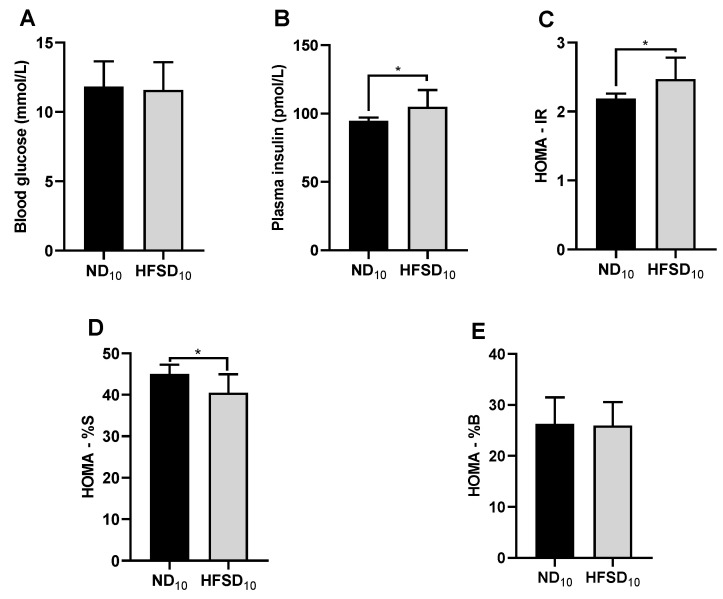
High-fat/sucrose diet feeding for 10 weeks causes dysregulated insulin metabolism. (**A**) Fasting blood glucose, (**B**) fasting plasma insulin, (**C**) insulin resistance, (**D**) insulin sensitivity, and (**E**) beta cell function in mice fed a normal diet or high-fat/sucrose diet for 10 weeks (*n* ≥ 6 per group). Data expressed as means ± SD. ND_10_—10-week normal diet; HFSD_10_—10-week high-fat/sucrose diet; * *p* ≤ 0.05.

After 10 weeks, normal diet and high-fat/sucrose diet-fed mice did not differ in fasting blood glucose (Figure 2A) but plasma insulin was increased (Figure 2B). However, the high-fat/sucrose diet caused hyperinsulinemia (Figure 2B), insulin resistance (Figure 2C) and reduced insulin sensitivity (Figure 2D).

### 2.2. High-Fat/Sucrose Feeding for 10 Weeks Impairs Corneal Wound Healing and Dysregulates Abrasion-Induced Inflammation

**Figure 3:**
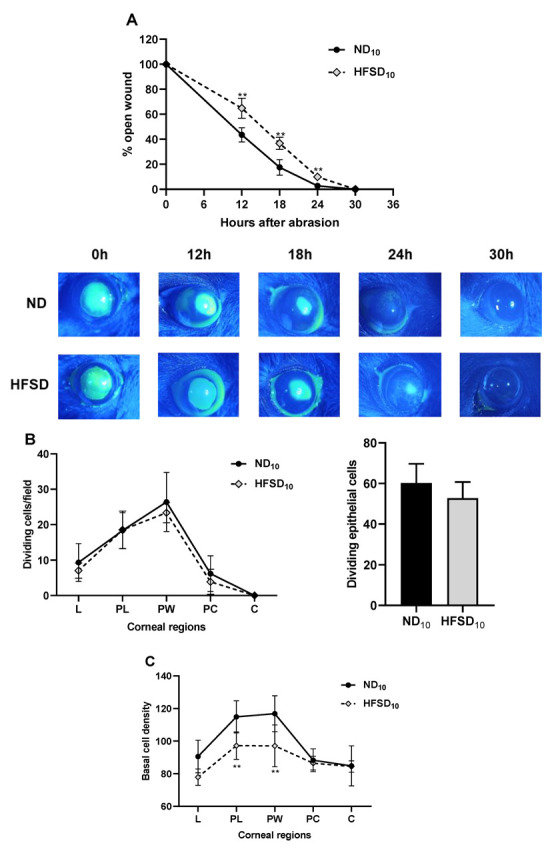
High-fat/sucrose diet feeding for 10 weeks impairs corneal wound healing. (**A**) Corneal wound closure in mice fed a normal or high-fat/sucrose diet. Top: Wound closure kinetics. Bottom: Representative images of the epithelial wound immediately after wounding, 12 h, 18 h, 24 h, and 30 h after wounding. (**B**) Left: Dividing basal epithelial cells in each region of the cornea. Right: Sum of dividing basal epithelial cells in four fields of view from the periphery to the center of the cornea & (**C**) Basal epithelial cell density in each region of the cornea at 30 h after wounding. Data expressed as mean ± S.D. ND_10_—10-week normal diet; HFSD_10_—10-week high-fat/sucrose diet; L—limbus; PL—paralimbus/periphery; PW—parawound; PC—paracentral; C—center; ** *p* ≤ 0.01.

In mice fed the normal diet for 10 weeks, wound closure following a 2 mm corneal epithelial abrasion wound was completed 24 h after wounding. Wound closure in mice fed the high-fat/sucrose diet for 10 weeks was delayed by ~6 h (Figure 3A). At 30 h post wounding, the number of dividing epithelial cells across the different corneal regions did not differ between the two groups (Figure 3B). Basal epithelial cell density is a sensitive indicator of re-epithelialization [32,33]. In the peripheral and parawound regions, basal epithelial cell density was significantly reduced in mice fed the high-fat/sucrose diet at 30 h post wounding (Figure 3C).

**Figure 4:**
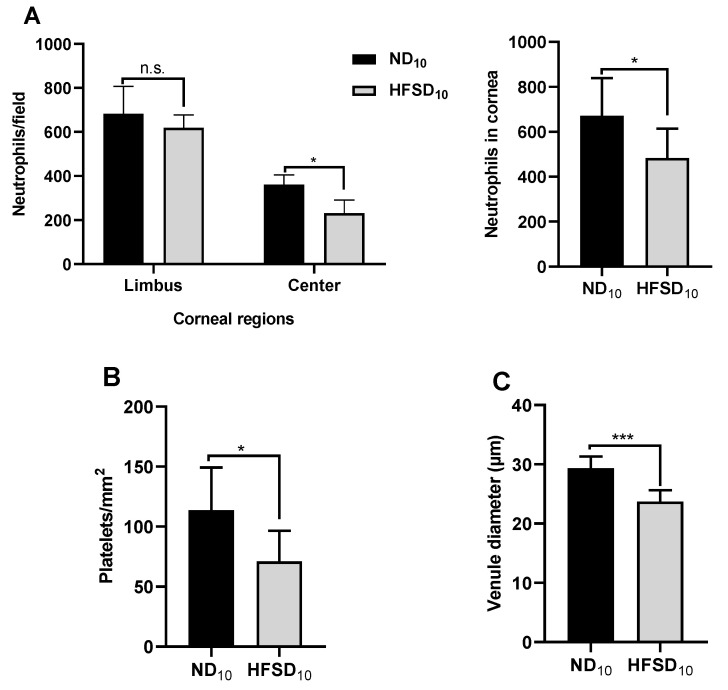
High-fat/sucrose diet feeding for 10 weeks dysregulates the inflammatory response of the cornea to abrasion when measured 30 h after wounding. (**A**) Left: The distribution of neutrophils over the limbal and central regions of the corneal stroma. Right: Sum of neutrophils in four fields of view from the periphery to the center of the cornea (*n* ≥ 6), (**B**) Platelet counts at the limbus (*n* ≥ 6). (**C**) Venule diameter at the corneal limbus of mice fed the normal or high-fat/sucrose diet. Data expressed as means ± SD. ND_10_—10-week normal diet; HFSD_10_—10-week high fat diet; n.s.—not significant; * *p* ≤ 0.05, *** *p* ≤ 0.001.

Regulated neutrophil extravasation and subsequent migration to the wound center are crucial for efficient corneal wound healing. At 30 h post-wounding, the number of extravasated neutrophils at the corneal limbus was not different between high-fat/sucrose diet-fed and normal diet-fed mice after the 10-week feeding. However, neutrophil migration to the wound center was impaired in mice fed the high-fat/sucrose diet, evidenced by the lower number of neutrophils at the wound center (Figure 4A). Additionally, the total number of neutrophils in the cornea was reduced (Figure 4A). Platelet extravasation (Figure 4B) and limbal vessel engorgement, measured by increased venule diameter (Figure 4C), were significantly reduced in mice fed the high-fat/sucrose diet.

### 2.3. Diet Reversal Attenuates High-Fat/Sucrose Diet-Induced Adiposity and Liver Weight

**Figure 5:**
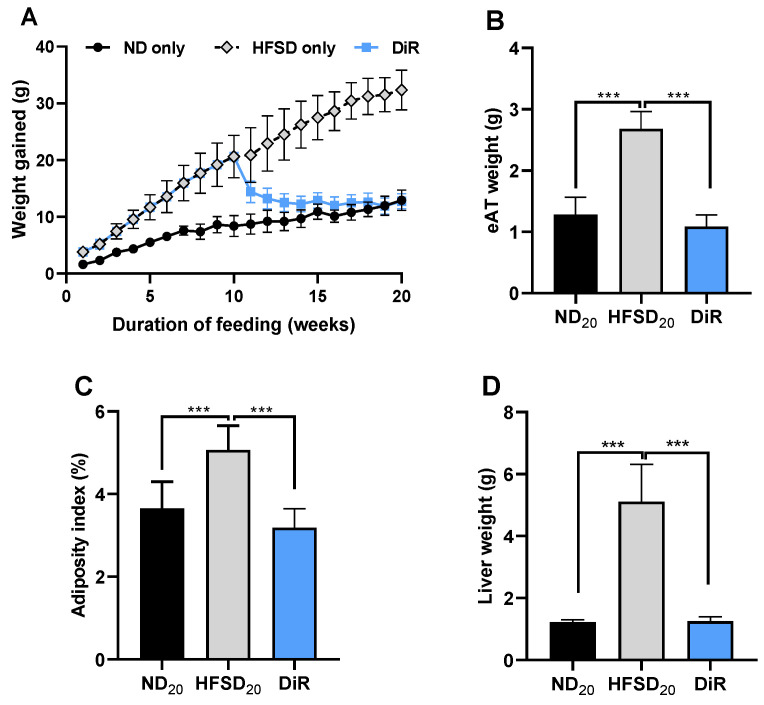
Diet reversal attenuates adiposity and liver weight gain. Mice were fed for a total of 20 weeks. (**A**) Cumulative weight change (**B**) Epididymal adipose tissue weight, (**C**) body adiposity index, and (**D**) liver weight in mice fed only the normal diet, only the high-fat/sucrose diet, and the diet reversal mice (*n* ≥ 7 per group). Data expressed as mean ± S.D. ND_20_—20-week normal diet; HFSD_20_—20-week high fat diet; DiR—diet reversal; *** *p* ≤ 0.001.

Mice fed only the high-fat/sucrose diet gained more weight than those fed only the normal diet. (Figure 5A). Switching mice from the high-fat/sucrose diet to the normal diet (diet reversal) resulted in a marked decrease in body weight. After 10 weeks of diet reversal, there was no difference in weight between the diet reversal mice and their age-matched normal diet-only mice (Figure 5A). Mice fed only the high-fat/sucrose diet for the full 20 weeks had significant visceral adiposity. Diet reversal attenuated visceral adiposity; epididymal adipose tissue weight and the adiposity index did not differ between the diet reversal mice and age-matched mice fed only the normal diet (Figure 5B,C). Increased liver weight was observed in mice fed only the high-fat/sucrose diet; this was ameliorated by diet reversal (Figure 5D).

**Figure 6:**
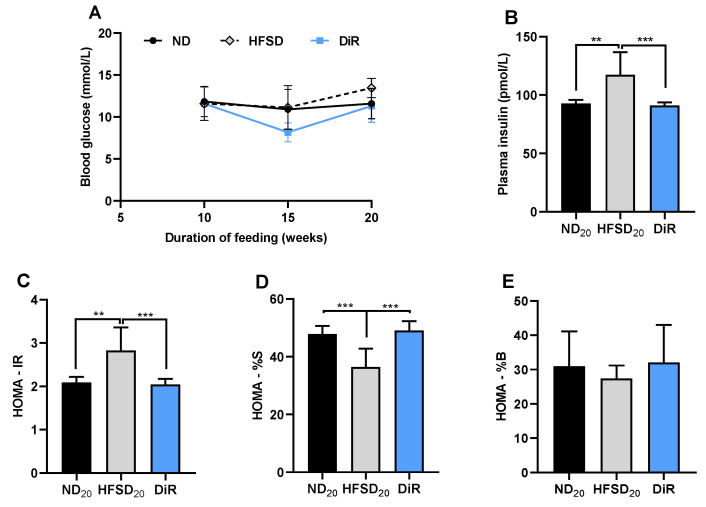
Diet reversal ameliorates dysregulated insulin sensitivity (**A**) Fasting blood glucose levels (*n* ≥ 7 per group). (**B**) Plasma insulin levels, (**C**) Insulin resistance, (**D**) Insulin sensitivity, and (**E**) Beta cell function in mice fed only the normal diet, only the high-fat/sucrose diet, and the diet reversal mice (*n* ≥ 7 per group). Data expressed as mean ± S.D. ND_20_—20-week normal diet; HFSD_20_—20-week high fat diet; DiR—diet reversal; ** *p* ≤ 0.01, *** *p* ≤ 0.001.

Mice fed on the normal diet and those fed only the high-fat/sucrose diet for 10 or 20 weeks did not differ in fasting blood glucose (Figure 6A). However, hyperinsulinemia (Figure 6B), insulin resistance (Figure 6C), and reduced insulin sensitivity (Figure 6D) previously observed at 10 weeks in mice fed the high-fat/sucrose diet persisted at 20 weeks. Diet reversal reduced plasma insulin concentration and insulin resistance and increased insulin sensitivity to levels comparable to age-matched mice fed only the normal diet (Figure 6B–D). Beta cell function did not differ between the three groups (Figure 6E).

### 2.4. Diet Reversal Halts Progression of Corneal Nerve Function Loss and Restored Normal Corneal Wound Healing

**Figure 7:**
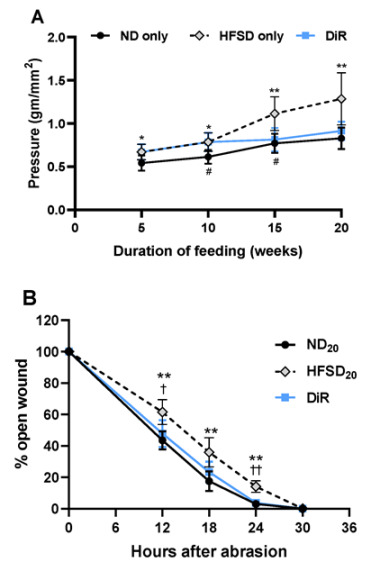

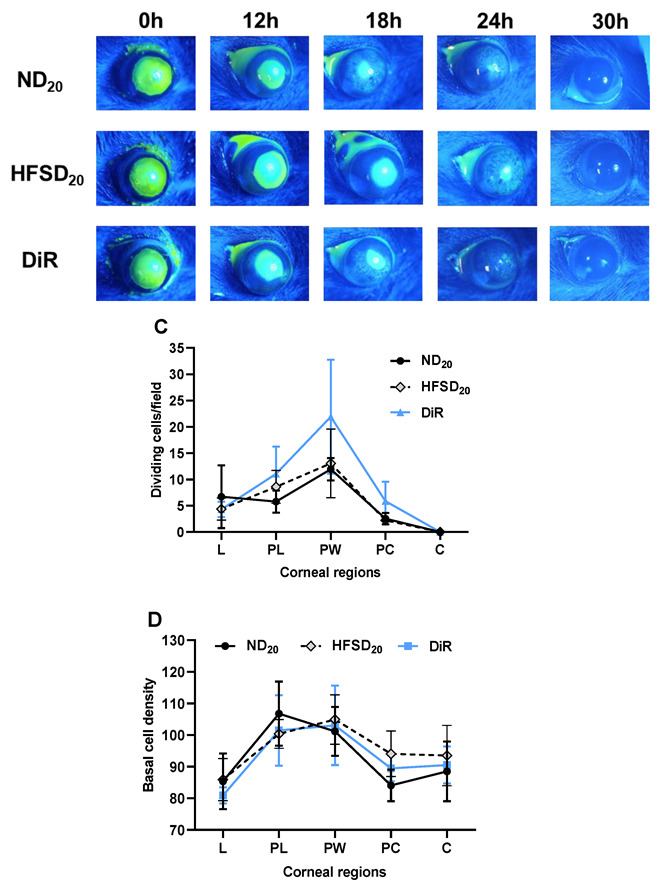
Diet reversal ameliorated impaired corneal wound healing and halted the progression of corneal nerve sensitivity loss observed with high-fat/sucrose consumption. (**A**) Corneal nerve sensitivity in uninjured mice and (**B**) Corneal wound closure Top: Wound closure kinetics. Bottom: Representative images of the epithelial wound at different time points following a 2 mm diameter corneal epithelial abrasion wound. (**C**) Left: Dividing basal epithelial cells in each cornea region. Right: Sum of dividing basal epithelial cells in four fields of view of the cornea (periphery, parawound, paracentral and center) and (**D**) Basal epithelial cell density in each cornea region in mice fed only the normal diet, only the high-fat/sucrose diet, and the diet reversal mice at 30 h post wounding. Data expressed as means ± SD; ND_20_—20-week normal diet; HFSD_20_—20-week high-fat/sucrose diet; DiR—diet reversal; L—limbus; PL—paralimbus/periphery; PW—parawound; PC—paracentral; C—center * *p* ≤ 0.05, ** *p* ≤ 0.01 (normal diet vs. high-fat/sucrose diet); † *p* ≤ 0.05 and †† *p* ≤ 0.01 (high-fat/sucrose diet vs. diet reversal); # *p* ≤ 0.05 (comparing corneal sensitivity in normal diet fed mice at different time points).

Previously, we reported the effect of a 5, 10, and 15-week high-fat/sucrose diet feeding on corneal nerve sensitivity [18]. The current study observed an age-related decline in corneal nerve sensitivity (measured as an increase in the filament pressure needed to elicit a blink when using the Cochet Bonnet aesthesiometer) in mice fed only the normal diet. (*p* < 0.05) Consistent with our earlier report, corneal nerve sensitivity was significantly reduced (expressed as more pressure to produce a blink) in mice fed only the high-fat/sucrose diet compared to those fed only the normal diet. The magnitude of corneal nerve sensitivity loss was dependent on the duration of feeding (Figure 7A). Delayed wound closure present at 12-, 18- and 24 h post-wound in mice fed a high-fat/sucrose diet for 10 weeks (Figure 3A) was also seen in mice fed the diet for 20 weeks (Figure 7B). Contrary to results at 10-week feeding, basal epithelial cell density was not different between the group fed only the normal diet and those fed only the high-fat/sucrose diet for 20 weeks (Figure 7D). Reversal from the high-fat/sucrose diet to the normal diet halted the progression of corneal nerve function loss and restored normal corneal wound closure (Figure 7A,B).

**Figure 8:**
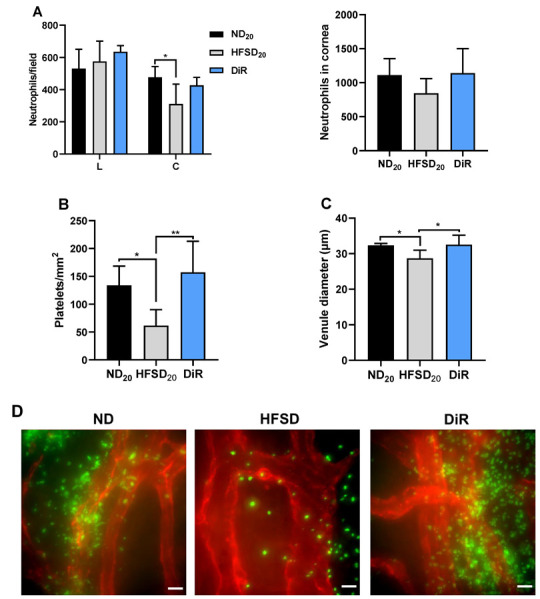
Diet reversal restored normal abrasion-induced inflammation. (**A**) Left: The distribution of neutrophils over the limbus and center of the cornea 30 h after wounding. Right: Sum of neutrophils in four fields of view of the cornea (periphery, parawound, paracentral and center) at 30 h after wounding. (**B**) Platelets count at the limbus 30 h after wounding. (**C**) Diameter of venules at the corneal limbus. (**D**) Representative images of platelets at the limbus; images captured with a 60X oil objective lens (scale bar = 15 µm). Data expressed as means ± SD; ND_20_—20-week normal diet; HFSD_20_—20-week high-fat/sucrose diet; DiR—diet reversal; L—limbus; C—center * *p* ≤ 0.05, ** *p* ≤ 0.01. Scale bars: D = 15 µm.

The impaired neutrophil migration to the center of the cornea and decreased platelet extravasation observed 30 h after wounding in mice fed a high-fat/sucrose diet for 10 weeks persisted after 20 weeks of feeding. The number of neutrophils at the center of the cornea and platelets at the limbus were significantly reduced (Figure 8A,B). Diet reversal restored neutrophil migration to the center of the cornea and platelet extravasation. The number of neutrophils at the corneal center and platelets at the limbus were comparable between mice fed only the normal diet and the diet reversal mice (Figure 8A,B). Venule engorgement following corneal abrasion, measured by venule diameter, was decreased in mice fed the high-fat/sucrose diet; diet reversal restored normal venule engorgement (Figure 8C). Figure 8D shows representative images of platelets at the limbus 30 h post wounding.

## 3. Discussion

The current study aimed to evaluate the effect of long-term high-fat/sucrose diet feeding on corneal wound healing and the utility of dietary macronutrient composition change for ameliorating these effects. We found that (1) a high-fat/sucrose diet causes significant weight gain, adiposity, hyperinsulinemia, insulin resistance and increased liver weight (2) a high-fat/sucrose diet significantly reduces corneal nerve function, impairs corneal wound healing, and dysregulates inflammation in response to corneal abrasion; (3) In addition to mitigating the cardiometabolic changes, diet reversal halted progression of corneal nerve function loss, ameliorated impaired corneal wound healing and dysregulated inflammatory response to corneal abrasion wound.

Mice fed the high-fat/sucrose diet (42% kcal fat & 30% kcal sucrose) developed significant adiposity, insulin resistance, hyperinsulinemia, and increased liver weight at 10 and 20 weeks of feeding. This mirrors human obesity and prediabetes [34,35,36]. These mice also develop corneal neuropathy, independent of sustained hyperglycemia, making this an ideal animal model to study diet-based therapeutic interventional strategies. Fat and sugar consumption drives the development of diet-induced obesity. Hence, reducing the fat and sugar content of diet while increasing complex carbohydrates and vegetables could be vital in treating obesity. In the current study, changing the macronutrient composition of diet reversed pre-existing adiposity, insulin resistance, hyperinsulinemia, and fatty liver. Other authors have reported similar results. Roberts et al. [37] reported that reversing rats from a high-fat and sugar diet to a low-fat, complex carbohydrate diet ameliorates obesity and normalizes glucose transport, plasma insulin, blood pressure, and low-density lipoprotein cholesterol. Parekh et al. reported a reversal of adiposity, hyperinsulinemia, and impaired glucose tolerance after switching mice from a high-fat diet (58% kcal/g fat) to a low-fat diet (11% kcal/g fat). Adiposity and fasting insulin and glucose values in mice switched from the high-fat diet to the low-fat diet were equivalent to those in mice that spent the entire experimental period on the low-fat diet. They concluded that obesity is entirely reversible through a reduction in dietary fat [38]. Sims-Robinson et al., using C57BL/6 mice, showed that high-fat diet feeding (54% kcal/g fat) impairs hippocampal insulin signaling and causes short- and long-term memory deficits. Reversing mice from the high-fat diet to a diet low in fat (10% kcal/g from fat) improved short-term and long-term memory [27]. Consumption of a high-fat diet disrupts the temporal coordination of circadian rhythm; the phase of the liver molecular clock is advanced, and daily rhythms of eating behavior and locomotor activity are altered [39,40]. Branechy et al. reported that changing the composition of diet from high fat to low fat restores the phase of the liver circadian clock and the rhythm of eating behavior [30].

In the current study, we observed delayed wound closure in mice fed the high-fat sucrose diet, at both 10 and 20 weeks of experimental feeding; wound closure was delayed by approximately 6 h. We had previously reported similar findings of impaired corneal wound healing after 10 days of high-fat/sucrose diet [18,19]. Obesity induced impaired corneal wound healing has been reported by other authors. Kang et al. using a diet-induced obesity mouse model reported impaired corneal wound healing after 6 weeks of high-fat diet feeding [41]. Well-regulated extravasation of neutrophils and platelets at the corneal limbus and subsequent migration of neutrophils to the center of the cornea is vital for corneal wound closure and re-epithelialization following a corneal epithelial abrasion wound. Consistent with our earlier reports at 10 days of high-fat/sucrose diet [18,19], high-fat/sucrose diet feeding for 10 and 20 weeks caused a dysregulated neutrophil and platelet influx into the cornea. The number of extravasated platelets at the limbus and neutrophils at the center of the cornea was reduced when measured at 30 h post wounding. Reversing mice to the normal diet, which is low in fat and sugar, restored normal corneal wound closure and the normal influx of platelet at the limbus and migration of neutrophils to the center of the cornea.

The effects of the high-fat/sucrose diet on corneal wound healing and inflammation and the restoration of normal dynamics of corneal wound healing following diet reversal may be explained by diet-induced gut dysbiosis and the significance of a healthy gut microbiome to corneal health and wound healing. The gut microbiome, the collection of microbes and their habitat, is vital to the metabolic effects of diet and metabolism, playing an essential role in body growth and immune development by controlling hormone secretion and modulating immune system maturation. The gut microbiome is unique and remains stable under normal physiological conditions after the first year of life. However, environmental factors such as diet composition and diet intake timing can cause gut microbiome dysfunction, known as gut dysbiosis [42,43,44]. Diet can restructure the gut microbiome within days and is reversible on a similar timescale [42,45]. The commonest bacteria phyla in the gut are Firmicutes (F) and Bacteroidetes (B). The ratio of Firmicutes to Bacteroidetes (F/B) is linked to health and disease. High-fat and high-sucrose diets feeding causes gut dysbiosis, decreasing Bacteroidetes while increasing Firmicutes, thus increasing the F/B ratio [46,47,48]. Increased F/B ratio favors weight gain, adiposity, and cardiometabolic complications such as insulin resistance, systemic inflammation, metabolic syndrome, diabetes, etc. High-fat/sucrose diet also reduces the diversity of the gut bacterial population and alters gene and metabolic pathway expression in these bacteria [49,50,51]. A high-fat/sucrose diet altered gut microbiome is rich in gene-encoding pathways related to fructose and mannose metabolism and glycolysis or gluconeogenesis but poor in those required for the metabolism of starch [51]. Diet-induced gut dysbiosis has been linked to impaired corneal wound healing and dysregulated abrasion-induced inflammation in diet-induced obesity. Kang et al. [41] showed that high-fat diet feeding causes gut dysbiosis, which is vital to obesity-induced impaired corneal wound feeding. Restoration of normal gut microbiome via the transplantation of fecal matter from mice fed a normal “healthy” chow diet to those fed the high-fat diet restored normal corneal wound healing. The authors thus concluded that high-fat diet-induced impaired corneal wound healing was due to diet-induced dysregulation of the gut microbial population. The experimental diet (high-fat/sucrose diet) used in the current study is high in saturated fatty acids and sucrose. Both saturated fatty acids and sucrose dysregulate the gut microbial population [46,47,48]. The control diet (normal chow diet) is low in saturated fatty acids and sucrose but high in complex carbohydrates. Rosas-Villegas et al., using an experimental design similar to that used in the current study, demonstrated that switching rats from a high-fat/sucrose diet to a control normal chow diet ameliorates pre-existing dysregulation of the gut microbiome and restores the normal gut microbial phylogeny [48]. Hence, we hypothesize that the amelioration of high-fat diet-induced cardiometabolic complications and restoring normal corneal wound healing after diet reversal is due to normal gut microbial phylogeny restoration.

We observed an age-related decrease in corneal nerve function. The effect of aging on corneal nerve function is well-reported in humans [52,53] and rodents [54]. High-fat/sucrose diet feeding exacerbated the age-related decrease in corneal nerve function in a duration of feeding-dependent fashion. This confirms our earlier report of reduced corneal nerve function in diet-induced obesity [18,19]. Reduced corneal nerve function and density in diet-induced obesity has been reported by other authors [15,18,29]. Confirming our earlier report [18] and report from other authors [55], changing the macronutrient composition of diet, through the diet reversal strategy, halted the progression of corneal nerve function loss. At the end of experimental feeding, the corneal nerve function in the mice fed only the normal diet and the diet reversal mice did not differ. Improvement in nerve function and peripheral neuropathy following changes in the macronutrient composition of diet has been reported in other tissues [56,57]. Rumora et al. reported that feeding C57BL/6 mice a diet high in saturated fatty acids (60% of total fat content) caused impaired sural and sciatic nerve conduction velocity. Switching the mice from a diet high in saturated fatty acids to a diet high in monounsaturated fatty acids restored nerve function and improved nerve conduction velocity [56]. The fatty acid composition of the diets may explain the neural benefits following the diet switch. The high-fat/sucrose diet used in the current study is high in saturated fatty acids, accounting for ~70% of the fat content compared to just 0.97% of saturated fatty acids in the normal diet. Peripheral nerves, such as the corneal nerves, utilize aerobic (mitochondrial-dependent) ATP production for normal nerve function. Mitochondria are produced in the cell body and distributed throughout the nerve structure using axonal transport systems [58,59]. Saturated fatty acids decrease the number and degree of motility of motile axonal mitochondria and induce mitochondrial depolarization [60,61]. This also impairs mitochondrial bioenergetics, causing dysfunctional ATP production and low intracellular levels of ATP, resulting in peripheral nerve apoptosis [61]. Unsaturated fatty acids, on the other hand, improve lipid homeostasis and mitochondrial function via upregulation of mitochondrial oxidative pathway genes, increasing mitochondria-dependent ATP production [62,63,64]. In peripheral nerves, unsaturated fatty acids, primarily monounsaturated fatty acids, restore mitochondrial membrane potential, improving transport mechanisms for distributing mitochondria throughout the nerve structure. Unsaturated fatty acids also reverse saturated fatty acids-induced mitochondrial depolarization and decreased ATP production, preventing nerve apoptosis [56].

The results from the current study may have translational implications for human obesity. Recent reports have shown the presence of corneal neuropathy in obese human subjects without the presence of sustained hyperglycemia [21,22]. Decreasing the saturated/increasing unsaturated fatty acid content and decreasing the sugar content of diet may be used as a low-cost and easily accessible strategy for treating corneal neuropathy and impaired corneal wound healing observed in obesity and other metabolic disorders. A study by Lewis et al. reported improved corneal neuropathy in patients with type 1 diabetes following treatment with seal oil omega-3 polyunsaturated fatty acid [65]. The findings from the current study and the report by Lewis et al. strongly support a diet-based interventional strategy for treating corneal neuropathy in obesity and prediabetes.

In summary, long-term high-fat/sucrose diet feeding causes reduced corneal nerve function, delayed corneal wound closure, and dysregulated inflammation, characterized by a reduced number of extravasated platelets at the limbus and neutrophils at the center of the cornea. In addition to reversing high-fat/sucrose diet-induced cardiometabolic changes, dietary composition change reverses pre-existing corneal dysregulation.

## 4. Materials and Methods

A catalog of materials and equipment used in this experiment is presented in Table S1.

### 4.1. Mice, Diet, and Experimental Design

Six-week-old C57BL/6 male mice (Jackson Laboratory, Bar Harbor, ME, USA) were housed in the University of Houston vivarium under temperature control and a 12:12 light-dark cycle environment. We used male mice only because they exhibit a well-described higher susceptibility to adipose tissue oxidative stress and inflammation [66], compared to female mice. Estrogen in female mice is thought to protect against adipose tissue inflammation and resultant insulin resistance [67,68]. This is also the case in other published mouse models of diet-induced obesity [34,69,70]. For the initial study, mice were randomly assigned to two groups: a control group (*n* = 7) fed a normal chow diet (14.85% kcal/g fat, 0% kcal/g sucrose & 64.04% kcal/g complex carbohydrates; 5V5R, LabDiet, St. Louis, MO, USA) ad libitum and an experimental group (*n* = 7) fed a high-fat/sucrose diet (41.31% kcal/g fat & 29.85% kcal/g sucrose; Diet #112734; Dyets Inc., Bethlehem, PA, USA) ad libitum for 10 weeks. For the diet reversal intervention, mice were fed either ad libitum normal diet (control group) or high-fat/sucrose diet. The control group remained on the normal diet for the whole study. After 10 weeks, some of the mice fed the high-fat/sucrose diet (*n* = 7) were switched to the normal diet for an additional 10 weeks (diet reversal group) while the rest continued the high-fat/sucrose diet for 10 more weeks. The high fat/sucrose diet closely mirrored the Western diet; both are high in saturated fats and sugar. This diet commonly known as the Western diet is extensively used in literature to generate diet-induced obesity mouse models [71,72,73].The Institutional Animal Care and Use Committee at the University of Houston approved the study (protocol #: 16-005). Procedures were performed according to the Association for Research in Vision and Ophthalmology Statement for the Use of Animals in Ophthalmic and Vision Research.

### 4.2. Body Adiposity and Fatty Liver

All mice were weighed at the start of experimental feeding and at weekly intervals for the duration of the study. At the end of the experiments, mice were euthanized by carbon dioxide inhalation followed by cervical dislocation. Epididymal adipose tissue, the fat mass surrounding the testes, was harvested and weighed. Body adiposity index, a measure of adiposity, was calculated as (epididymal adipose tissue weight/final body weight) × 100. The liver was also harvested and weighed.

### 4.3. Fasting Blood Glucose and Plasma Insulin

Before blood sampling via the tail vein for glucose measurements, mice were fasted for 5 h. Blood glucose was measured at 10, 15, and 20 weeks of experimental feeding using a OneTouch Ultra glucose meter (LifeScan, Milpitas, CA, USA). The OneTouch Ultra glucose meter is routinely used to measure mouse blood glucose levels [69,74,75]. At terminal time points, mice were fasted for 5 h and anesthetized prior to blood collection via cardiac puncture using K2EDTA (SAI Infusion Technologies, Lake Villa, IL, USA) as an anticoagulant. Anesthesia was achieved by intraperitoneal injection of a ketamine (80 mg/Kg body weight) and xylazine (8 mg/Kg body weight) mixture (Vedco, Inc., St. Joseph, MO, USA). Blood was centrifuged at 1000× *g* for 5 min. The supernatant (plasma) was aliquoted and stored at −80 °C for subsequent plasma insulin determinations using a mouse insulin enzyme-linked immunosorbent assay (ELISA) kit and following the manufacturer’s protocol (Thermo Fisher Scientific, Waltham, MA, USA). Insulin resistance (IR), % insulin sensitivity (%S), and % pancreatic beta cell function (%B) were estimated using the Homeostasis Model Assessment—2 (HOMA-2) index calculator from the Diabetes Trial Unit of the University of Oxford (HOMA2 Calculator: Overview (ox.ac. U.K.)). The HOMA-2 calculator uses blood glucose and plasma insulin as inputs. The HOMA-2 index is used experimentally to calculate IR, %S, and %B in rodents [70,76,77].

### 4.4. Corneal Nerve Function

Corneal nerve function was measured at 5, 10, 15, and 20 weeks of experimental feeding using a Cochet-Bonnet aesthesiometer (Richmond Products, Albuquerque, NM, USA). The monofilament of the aesthesiometer was held perpendicular to and pressed against the central cornea. The measurement began with the monofilament extended to its maximum length (6.0 cm) and then systematically decreased (0.5 cm increment) until a blink was observed. Filament pressure increases exponentially as filament length decreases. The filament pressure required to elicit a blink was estimated from a filament length/pressure chart supplied by the manufacturer.

### 4.5. Corneal Wound Healing

Under anesthesia, the center of the cornea was marked with a 2 mm diameter trephine (Integra, Princeton, NJ, USA), and the epithelium within the demarcated region was debrided with a blunt golf club spud (Stephens instruments, Lexington, KY, USA). Wounding was performed every morning (between 8 am and 12 pm) to avoid confounding circadian effects on wound healing and the inflammatory response. Anesthesia was achieved via intraperitoneal injection of a ketamine/xylazine cocktail (80 mg/8 mg/kg body weight). At the time of wounding (0 h) and 12-, 18-, 24-, and 30 h post-wounding, 1 μL of 1% sodium fluorescein solution (Sigma Aldrich, St. Louis, MO, USA) was instilled on the wound, and images of the open wound were captured with a stereomicroscope equipped with a digital camera and blue light illumination. The wound area was then measured with ImageJ software (NIH, Bethesda, MD, USA). Results for each time point were expressed as a percentage of the original wound size. The method for creating and monitoring the corneal abrasion wound has been described previously in detail [78].

### 4.6. Immunofluorescence Staining

Mice were euthanized 30 h after wounding, a timepoint that correlates with peak neutrophil infiltration into the cornea during wound healing [33]. The eyes were enucleated and fixed in phosphate-buffered saline (PBS; ThermoFisher Scientific, Waltham, MA, USA) containing 2% paraformaldehyde (Tousimus Research Corporation, Rockville, MD, USA) for 45 min at room temperature. Corneas were then excised from the eyeball, and permeabilized in PBS containing 2% bovine serum albumin (BSA; ThermoFisher Scientific, Waltham, MA, USA) and 0.01% TritonX-100 (Fisher Scientific, Waltham, MA, USA) for 15 min followed by blocking in PBS containing 2% BSA for an additional 45 min at room temperature. Corneas were then incubated overnight at 4 °C in a cocktail of fluorescently labeled antibodies (5–10 µg/mL) against neutrophils (anti-Ly6G; BD Pharmingen, San Diego, CA, USA), platelets (anti-CD41; BioLegend, San Diego, CA, USA) and limbal blood vessel endothelium (anti-CD31; BioLegend, San Diego, CA, USA). In addition, DAPI (4′,6-diamidino-2-phenylindole, Sigma-Aldrich, St. Louis, MO, USA) was added to the cocktail to visualize nuclei and mitotic figures. Corneas were flat mounted on a microscope slide in Airvol (Celanese, Dallas, TX, USA) and imaged with a DeltaVision epifluorescence light microscope (G.E. Life Sciences, Pittsburg, PA, USA). Full-thickness images were captured with a 30X silicon lens with an image size of 381 µm × 381 µm and a z-section thickness step size of 0.5 µm.

### 4.7. Morphometric Analysis of Neutrophil and Platelet Recruitment and Blood Vessel Diameter

Mitotic figures, used to assess epithelial cell division, were visualized via DAPI staining and neutrophils were visualized using DAPI and Ly-6G staining. To assess neutrophil infiltration and epithelial cell division, images were taken in the wound center and at the paracentral, parawound, periphery, and limbal regions of the cornea in each quadrant, as previously reported [33,78,79]. Neutrophil and mitotic figures counted in each region from the 4 petals except the center (which had counts from only one field per cornea) were averaged and expressed as counts per field. For extravascular platelet assessment, the entire corneal limbus was imaged in each petal. A maximum intensity projection was applied to merge the z-stack images and platelets were counted in each of the four petals and then summed together. The limbal area was measured by drawing a closed loop around the limbal vessels in each petal. Extravascular platelet counts were then expressed as platelets/mm^2^ of the limbal area since extravascular platelets are non-motile and remain within the limbus [80]. The same image projection was used to measure vessel diameter.

### 4.8. Statistical Analysis

GraphPad Prism 6 (GraphPad Software, La Jolla, CA, USA) was used for data analysis. Data were summarized as means ± standard deviations. Unpaired t-tests and ANOVAs (one-way, two-way, and repeated measures with Tukey post hoc tests for multiple comparisons) were used to analyze data when appropriate. For all statistical analyses, an alpha level of ≤0.05 was considered significant.

## Data Availability

The data presented in this study are available on request from the corresponding author.

## Supplementary Materials

The following supporting information can be downloaded at: https://www.mdpi.com/article/10.3390/ijms24020931/s1.
