# Supplementary material for: A Low-Fat/Sucrose Diet Rich in Complex Carbohydrates Reverses High-Fat/Sucrose Diet-Induced Corneal Dysregulation
Source: Int J Mol Sci. 2023 Jan 4;24(2):931. doi: 10.3390/ijms24020931 (PMC9865780; doi:10.3390/ijms24020931)
Supplement: Supplementary file 1 [file ijms-24-00931-s001.zip › ijms-2083217-supplementary.pdf]

Table S1 Materials and equipment used in experiment

Name of Material/ Equipment	Company	Catalog/Product Number
Anti-CD31 antibody	BD Bioscience, Pharmingen	550274
Anti-CD41 antibody	BD Bioscience, Pharmingen	553847
Anti-Ly6G antibody	BD Bioscience, Pharmingen	551459
Bovine serum albumin (BSA)	ThermoFisher scientific	B14
Cochet-Bonnet aesthesiometer	Richmond Products	
C57BL/6 mice	Jackson Laboratories	664
DAPI	Sigma Aldrich	D8417
Golf-club spud	Stephens instruments	S2-1135
ImageJ software	National Institute of Health	
Isoflurane	Patterson veterinary	07-893-1389
Ketamine	Vedco	VINV-KETA-0VED
Mouse insulin ELISA kit	Thermo Fisher Scientific	EMINS
OneTouch Ultra glucose meter	LifeScan	
Phosphate buffered saline (PBS)	ThermoFisher scientific	AM9624
Sodium fluorescein salt	Sigma Aldrich	46970
Trephine	Integra Miltex	33-31
TritonX -100	Fisher Scientific	50-295-34
Xylazine	Vedco	
